# P-1327. Clinical Outcomes In Patients Who Receive Ertapenem Vs. Meropenem For Extended Spectrum Beta-Lactamase (ESBL) Infections And Have Hypoalbuminemia

**DOI:** 10.1093/ofid/ofaf695.1515

**Published:** 2026-01-11

**Authors:** Kendall Ferrara, Melissa George, Josh Rumph

**Affiliations:** ECU Health Medical Center, Greenville, NC; ECU Health Medical Center, Greenville, NC; Novant Health, Winston-Salem, North Carolina

## Abstract

**Background:**

The standard of care for treatment of ESBL infections is a carbapenem. Ertapenem is highly protein bound. In patients that are hypoalbuminemic and/or critically ill, this leads to an increased free fraction of ertapenem and a significantly decreased half-life. Limited clinical data has shown a significantly increased risk of mortality, readmission, and length of stay in patients who are hypoalbuminemic and receive ertapenem compared to other carbapenems for ESBL infections. Based on this data, the Infectious Diseases Society of America suggests utilizing meropenem or imipenem-cilastatin in patients that are critically ill and/or hypoalbuminemic with ESBL infections outside of the urinary tract. Historically at East Carolina University Health, ertapenem has been more commonly used than meropenem for ESBL infections regardless of clinical status or albumin. This study aims to evaluate the treatment failure of ertapenem compared to meropenem in patients who have ESBL infections and hypoalbuminemia.

**Table 1.**
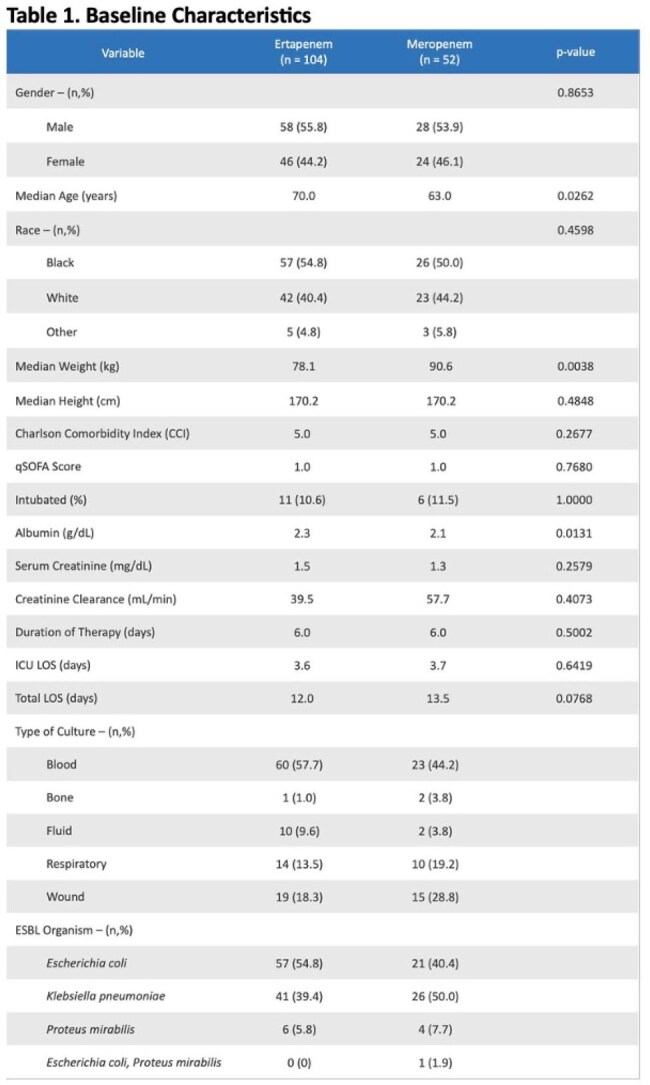
Baseline Characteristics

**Table 2.**
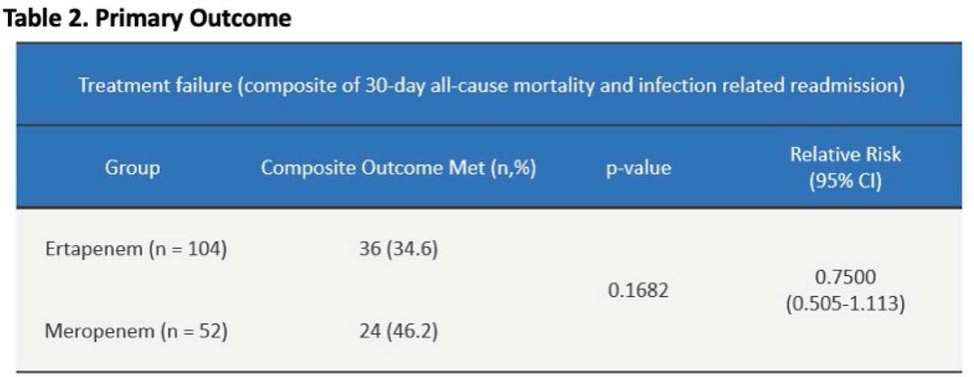
Primary Outcome

**Methods:**

This multicenter, retrospective chart review included hospitalized adult patients who received either ertapenem or meropenem from January 2008 to December 2024 with an albumin ≤2.5 g/dL within 72 hours before or after initiation of therapy. The primary endpoint was treatment failure, defined as a composite of 30-day all-cause mortality and infection related readmission. Secondary outcomes included the individual components of the primary outcome, 90-day all-cause mortality and infection related readmission, and hospital length of stay.

**Results:**

A total of 156 patients were included in the study (ertapenem n=104; meropenem n = 52). Baseline characteristics were similar overall between groups. Patients in the meropenem group had significantly lower albumin levels, but this was deemed to not be clinically significant. The most common type of culture in both groups was blood. *Escherichia coli* was the most frequently isolated organism (Table 1). There were no significant differences in the primary outcome (Table 2) or secondary outcomes.

**Conclusion:**

In patients with ESBL infections and hypoalbuminemia, there was no significant difference in treatment failure between ertapenem and meropenem. Larger, randomized, prospective studies are needed to confirm these findings.

**Disclosures:**

All Authors: No reported disclosures

